# Molecular Control of Oil Metabolism in the Endosperm of Seeds

**DOI:** 10.3390/ijms22041621

**Published:** 2021-02-05

**Authors:** Romane Miray, Sami Kazaz, Alexandra To, Sébastien Baud

**Affiliations:** Institut Jean-Pierre Bourgin, INRAE, CNRS, AgroParisTech, Université Paris-Saclay, 78000 Versailles, France; romane.miray@inrae.fr (R.M.); sami.kazaz@inrae.fr (S.K.); alexandra.to@inrae.fr (A.T.)

**Keywords:** seed, endosperm, oil, fatty acid, metabolism

## Abstract

In angiosperm seeds, the endosperm develops to varying degrees and accumulates different types of storage compounds remobilized by the seedling during early post-germinative growth. Whereas the molecular mechanisms controlling the metabolism of starch and seed-storage proteins in the endosperm of cereal grains are relatively well characterized, the regulation of oil metabolism in the endosperm of developing and germinating oilseeds has received particular attention only more recently, thanks to the emergence and continuous improvement of analytical techniques allowing the evaluation, within a spatial context, of gene activity on one side, and lipid metabolism on the other side. These studies represent a fundamental step toward the elucidation of the molecular mechanisms governing oil metabolism in this particular tissue. In particular, they highlight the importance of endosperm-specific transcriptional controls for determining original oil compositions usually observed in this tissue. In the light of this research, the biological functions of oils stored in the endosperm of seeds then appear to be more diverse than simply constituting a source of carbon made available for the germinating seedling.

## 1. Introduction: Seed Development and Oil Storage

In angiosperms, seeds originate from the fertilized ovule, with double fertilization of the embryo sac initiating the development of the embryo and the endosperm, which grow embedded in maternal tissues called seed coat [1,2]. From an evolutionary perspective, the origin of the endosperm remains debated [3,4,5]. A first hypothesis proposes that in ancient seeds or seed-like structures, the central cell used to be an additional gamete besides the egg cell. Two embryos may have been produced in the ancestors of flowering plants, one of which acquired a novel nourishing function to the benefit of the embryo proper during angiosperm evolution [6]. The second hypothesis suggests that the endosperm represents a homolog of the gymnosperm female gametophyte that became sexualized [7,8].

The relative contribution of the endosperm to the final mass of the mature seed varies considerably from one species to another [9,10,11]. Endosperm can be absent, as in Podostemaceae [12], where double fertilization is invariably missing. It may consist of one to a few peripheral layers of living cells surrounding a reserve-containing embryo, as in several Brassicaceae or Fabaceae species [13,14,15]. In contrast, endosperm constitutes most of the seed volume in the caryopsis of cereals and consists of abundant dead storage tissues called starchy endosperm that are surrounded by the living aleurone layer [16]. Most cereal grains contain a single-cell layered aleurone; *Hordeum vulgare* (barley) is an exception to this rule, with the aleurone comprising two to three cell layers [17].

The development of the endosperm can be divided into several phases conserved among most angiosperms [18]. After fertilization of the central cell, the initial endosperm nucleus divides repeatedly without cell-wall formation, resulting in a characteristic coenocytic-stage endosperm [19]. Once the nuclear division phase is complete, the multinucleate homogeneous cytoplasm reorganizes into nucleocytoplasmic domains, and the cellularization phase begins via the formation of radial microtubule systems and alveolation [20]. In cereal grains, periclinal cell divisions of the cell layer thus obtained give rise to two cell types: the outer aleurone and inner starchy endosperm initials. Endosperm cells then differentiate and accumulate reserve compounds during the maturation phase [21]. The starchy endosperm of cereal grains is subjected to programmed cell death during seed maturation, while the outer aleurone layer remains viable in the mature seed [22]. In seeds of many dicot species, the endosperm is gradually depleted as the embryo grows during early seed maturation, leaving only a peripheral aleurone-like cell layer in mature seeds.

Depending on the species considered, the main storage compounds accumulated in the endosperm consist of seed storage proteins, carbohydrates like starch or ß-glucans, or storage lipids like waxes or triacylglycerols [23,24]. Triacylglycerols are the most common storage lipids. They are composed of three fatty acyl groups esterified to a glycerol backbone at the *sn*-1, *sn*-2, and *sn*-3 positions. Triacylglycerols are accumulated in the endosperm of dicot oilseeds, regardless of the relative proportion of this endosperm in the seed, as in the single-layer aleurone-like endosperm of *Brassica napus* (rapeseed) or in the enlarged endosperm of *Ricinus communis* (castor) [25]. Triacylglycerols are also abundant in the large endosperm structure found in seeds of non-Poaceae monocots like *Elaeis guineensis* (oil palm) [26] or *Cocos nucifera* (coconut) [27]. Finally, while starch represents the main form of carbon storage in the starchy endosperm of most cereal grains, triacyglycerols appear to be preponderant in the aleurone layer of the very same grains [28]. While the regulation of starch metabolism in the endosperm of cereal grains has been thoroughly characterized [29,30], the study of lipid metabolism in endosperm tissues has only emerged more recently.

## 2. Oils Stored in the Endosperm Usually Differ from That Stored in the Embryo

### 2.1. Fatty Acid and Oil Biosynthesis in Seeds

**Figure 1:**
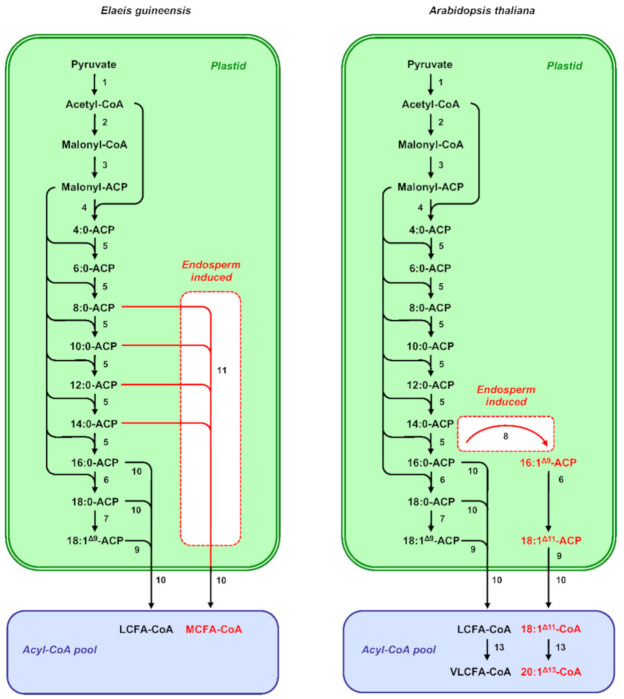
Overview of fatty acid metabolism in maturing seeds of *Elaeis guineensis* and *Arabidopsis thaliana*. The scheme depicts the different pathways involved in the synthesis and elongation of fatty acids in maturing seeds. Enzymatic steps transcriptionally induced in the endosperm with respect to the embryo are denoted in red and highlighted in white boxes. 1, pyruvate dehydrogenase; 2, acetyl-coenzyme A carboxylase; 3, malonyl-coenzyme A:acyl carrier protein S-malonyltransferase; 4, fatty acid synthase complex comprising 3-ketoacyl-ACP synthase III; 5, fatty acid synthase complex comprising 3-ketoacyl-ACP synthase I; 6, fatty acid synthase complex comprising 3-ketoacyl-ACP synthase II; 7, Δ^9^ stearoyl-acyl carrier protein desaturase; 8, Δ^9^ palmitoyl-acyl carrier protein desaturase; 9, FatA fatty acyl-ACP thioesterase; 10, FatB fatty acyl-ACP thioesterase; 11, Specialized FatB fatty acyl-ACP thioesterase releasing medium-chain fatty acids; 12, long-chain acyl-coenzyme A synthetase; 13, fatty acid elongase complex. Abbreviations: ACP, acyl carrier protein; CoA, coenzyme A; LCFA, long-chain fatty acid; VLCFA, very-long-chain fatty acid.

Plant de novo fatty-acid synthesis occurs in the plastids [24,31,32] (Figure 1). Formation of malonyl-coenzyme A (CoA) from acetyl-CoA and bicarbonate by acetyl-CoA carboxylase is considered as the first committed step of the pathway. The malonyl-CoA produced by acetyl-CoA carboxylase constitutes the carbon donor for each cycle of the fatty-acid biosynthesis pathway. Before entering the biosynthetic process, the malonyl group of malonyl-CoA has to be transferred from CoA to a protein cofactor named acyl carrier protein (ACP) by a malonyl-CoA: acyl carrier protein S-malonyltransferase. Production of saturated fatty acids is performed in a stepwise manner by the fatty-acid synthase of type II, which uses acetyl-CoA as a starting unit and malonyl-ACP as the elongator. Saturated acyl chains bound to ACPs can then be desaturated by stromal soluble acyl-ACP desaturases (AADs) to form *cis*-monoenes. Depending on their regiospecificity and substrate specificity, AADs can synthesize different types of *cis*-monoenes [33]. Δ^9^ Stearoyl-ACP desaturases (SADs) efficiently desaturate 18:0 to form 18:1^Δ9^ and represent the archetypal AAD [34,35]. However, variant-specialized AAD isoforms have been described in seeds of certain plant species that produce other types of monounsaturated fatty acids [36]. For example, Δ^9^ palmitoyl-ACP desaturases (PADs) prefer 16:0 as substrate, and catalyze the formation of 16:1^Δ9^ [37,38]. The synthesis of fatty acids in the plastids is terminated when the acyl group is removed from ACP by specific acyl-ACP thioesterases. Two classes of acyl-ACP thioesterases, termed FatA and FatB, have been described. Enzymes of the FatA class preferentially hydrolyze 18:1^Δ9^-ACP, whereas saturated acyl-ACP are the preferential substrate of FatB thioesterases [39]. The premature cleavage of saturated acyl chains from ACP can also be operated by additional specialized FatB thioesterases, yielding medium-chain fatty acids [40,41].

**Figure 2:**
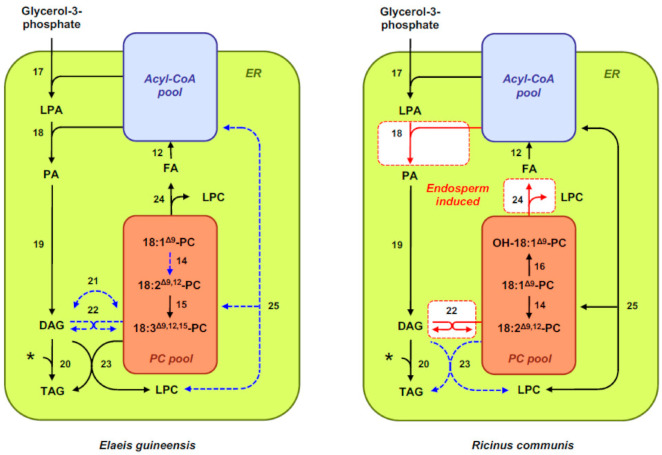
Overview of oil metabolism in maturing seeds of *Elaeis guineensis* and *Ricinus communis*. The scheme depicts the different pathways involved in the assembly of storage lipids in maturing seeds. Enzymatic steps transcriptionally induced in the endosperm with respect to the embryo are denoted in red and highlighted in white boxes. Blue dashed arrows denote enzymatic steps transcriptionally repressed in the endosperm. 12, long-chain acyl-coenzyme A synthetase; 14, Δ^12^ fatty acid desaturase; 15, Δ^15^ fatty acid desaturase; 16, Δ^12^ fatty acid hydroxylase; 17, acyl-coenzyme A:*sn*-glycerol-3-phosphate acyltransferase; 18, acyl-coenzyme A:lysophosphatidic acid acyltransferase; 19, phosphatidic acid phosphohydrolase; 20, acyl-coenzyme A:1,2-diacyl-*sn*-glycerol acyltransferase; 21, CDP-choline:1,2-*sn*-diacylglycerol choline phosphotransferase; 22, phosphatidylcholine:1,2-*sn*-diacylglycerol choline phosphotransferase; 23, phospholipid:1,2-*sn*-diacylglycerol acyltransferase; 24, phospholipase A_2_; 25, acyl-coenzyme A: lysophosphatidylcholine acyltransferase. Abbreviations and symbols: *, acyl-CoA; CoA, coenzyme A; DAG, diacylglycerol; ER, endoplasmic reticulum; FA, fatty acid; LPA, lysophosphatidic acid; LPC, lysophosphatidylcholine; PA, phosphatidic acid; PC, phosphatidylcholine; TAG, triacylglycerol; VLCFA, very-long-chain fatty acid.

After export from the plastid, activation to CoA esters, and transport to the endoplasmic reticulum (ER), these fatty acids can be further modified. A membrane-bound multienzyme complex called the fatty-acid elongase complex can elongate long-chain fatty acids to form very-long-chain fatty acids (C > 18). As for polyunsaturated fatty acids commonly found in seed oils, they are synthesized by membrane-bound desaturases (FADs) after incorporation of 18:1^Δ9^ into membrane phosphatidylcholine (PC) (Figure 2). The Δ^12^ fatty-acid desaturase FAD2 desaturates 18:1^Δ9^-PC into 18:2^Δ9,12^-PC [42], which can be further desaturated into 18:3^Δ9,12,15^-PC by the Δ^15^ fatty acid desaturase FAD3 [43]. Desaturases homologs to FAD2 have been described in seeds of certain plant species that exhibit a Δ^12^ fatty acid hydroxylase activity and produce hydroxylated fatty acids like ricinoleic acid (Δ^12^-OH-18:1^Δ9^) [44].

Triacylglycerol assembly takes place in the ER and involves multiple pathways interconnected with membrane-lipid biosynthesis pathways (Figure 2) [45]. Acyl-CoAs are used for the sequential acylation of glycerol-3-phosphate backbones produced from dihydroxyacetone phosphate by glycerol-3-phosphate dehydrogenase. The first acylation is catalyzed by acyl-CoA:*sn*-glycerol-3-phosphate acyltransferase (GPAT), yielding lysophosphatidic acid (LPA) [46]. The second acylation, catalyzed by acyl-CoA:lisophosphatidic acid acyltransferase (LPAAT), produces phosphatidic acid (PA) [47]. PA is then converted to 1,2-*sn*-diacylglycerol (DAG) by phosphatidic acid phosphatases [48]. DAG represents a branch point between triacylglycerol and membrane-lipid synthesis. In the straightforward Kennedy pathway, a third acyl-CoA is used by 1,2-*sn*-diacylglycerol acyltransferases (DGATs) to directly form triacylglycerol. However, studies have shown that DAG originating from the Kennedy pathway is efficiently converted to PC and then reconverted back to DAG before being channeled to triacylglycerol. This flux allows the incorporation of PC-modified fatty acids into triacylglycerol molecules [49]. DAG can be converted into PC by the reversible reaction catalyzed by CDP-choline:1,2-*sn*-diacylglycerol choline phosphotransferase (CPT). Phosphatidylcholine:1,2-*sn*-diacylglycerol choline phosphotransferase (PDCT), which catalyzes the transfer of a phosphocholine head group between DAG and PC, provides another route for desaturated acyl chains from the PC pool to be returned to the DAG pool [50]. Finally, acyl-editing mechanisms represent another possibility for moving polyunsaturated or unusual fatty acids from PC to the acyl-CoA pool, where these acyl chains are made available for the acyl-CoA-dependent acyltransferases of the Kennedy pathway. An acyl-editing cycle called the Lands cycle involves PC-deacylation and lysophosphatidylcholine (LPC)-reacylation cycles, and allows exchanges of fatty acids between PC and the acyl-CoA pool without net PC synthesis or degradation [51]. In this mechanism, a phospholipase A_2_ hydrolyzes acyl groups at the *sn*-2 position of PC, producing LPC and fatty acids available for subsequent activation by long-chain acyl-CoA synthetase. Reacylation of LPC is mediated by acyl-CoA:lysophosphatidylcholine acyltransferase (LPCAT), producing a PC molecule different in fatty acyl chains [52]. Once triacylglycerol assembly is achieved, possibly in specialized subdomains of the ER, triacylglycerol molecules are transferred and accumulated into spherical organelles called lipid droplets, oil bodies, or oleosomes. These organelles comprise a matrix of triacylglycerols surrounded by a phospholipid monolayer [53], into which a specific subset of proteins is embedded [54]. Oleosins, the most abundant proteins in this monolayer, cover the entire surface of oil bodies, preventing coalescence or aggregation of these organelles in mature seeds [55].

### 2.2. Contrasted Oil Contents in the Different Tissues of the Seed

**Figure 3:**
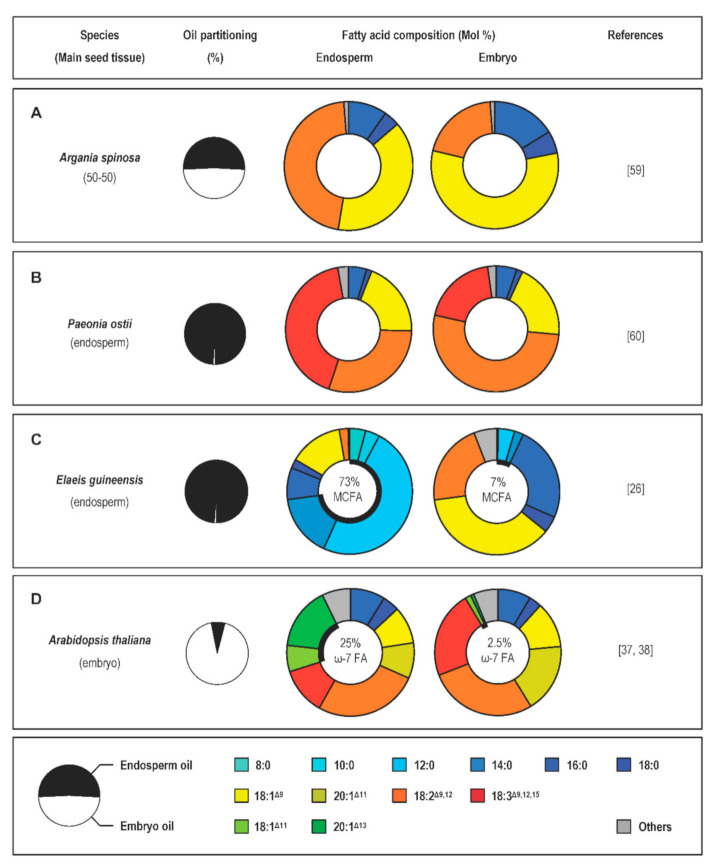
Fatty acid composition of endosperm and embryo oils in seeds of different species. (**A**). *Argania spinosa*. (**B**). *Paeonia ostii*. (**C**). *Elaeis guineensis*. (**D**). *Arabidopsis thaliana*. For each species considered, a pie chart presents the repartition of seed oil between embryo and endosperm tissues. Circular charts present the relative proportions of the main fatty acid species comprising endosperm and embryo oils, respectively. The relative abundance of unusual fatty acids in each compartment is indicated in the center of the circular charts. FA, fatty acid; MCFA, medium-chain fatty acid.

Angiosperms collectively display huge variation in the fatty acids they store in seeds [24]. Meticulous accumulation of datasets from several decades of research has led to the development of the Seed Oil Fatty Acid (SOFA) database and the PlantFAdb website, which displays over 450 different fatty-acid structures and seed fatty-acid composition data for over 9000 plants [56,57,58]. In contrast with the abundance of data describing the overall fatty-acid composition of seeds, reports describing the separate analysis of the different seed tissues accumulating oil are far less common. Manual dissection followed by gas chromatography analysis of seed fractions has revealed very contrasted situations, depending on the species considered. If similar ranges of fatty-acid species are usually observed in the two seed compartments of most seeds analyzed, contrasted proportions of these fatty-acid species in the two zygotic tissues were often reported. In oleaginous seeds of *Argania spinosa* (argan), the respective sizes and oil contents of the embryo and endosperm tissues are in the same order of magnitude. However, contrasting fatty-acid compositions were described in these two tissues [59]. If palmitic (16:0), stearic (18:0), oleic (18:1^Δ9^), and linoleic (18:2^Δ9,12^) acids are the major fatty acids in both tissues, the relative proportion of 18:2^Δ9,12^ in the endosperm is twofold higher than that of the embryo (Figure 3A). In albuminous seeds of *Paeonia ostii* and *Paeonia rockii*, 16:0, 18:1^Δ9^, 18:2^Δ9,12^, and 18:3^Δ9,12,15^ represent the major fatty acids present in both endosperm and embryo oils, but the relative proportion of 18:3^Δ9,12,15^ in the endosperm is twofold higher than that of the embryo (Figure 3B) [60].

In some oleaginous species, the endosperm exhibits high levels of unusual fatty acids. Seeds of *E. guineensis* comprise a copious endosperm and a small embryo accounting for 0.4% of the mature seed dry mass [26]. In mature embryos, lipid droplets are present at the periphery of the cells. They represent 27% of the dry mass and display high levels of 18:1^Δ9^, 16:0, and 18:2^Δ9,12^. In contrast, the endosperm of palm oil is well known for its exceptionally high oil content (approximately 50% of the dry mass), with lipid droplets occupying the majority of the cell volume. Medium-chain fatty acids like 12:0 (50 Mol%), 14:0, 8:0, and 10:0 predominate in endosperm oil, accounting for roughly 75 Mol% of total fatty acids (Figure 3C). A very similar situation was observed in seeds of *C. nucifera*, another species in the family Arecaceae [61,62]. Embryo tissues are relatively poor in lipids and their major fatty-acid components are 18:2^Δ9,12^, 16:0, and 18:1^Δ9^. In contrast, the endosperm accumulates high amounts of oil (the fatty-acid content in mature tissues represents about 40% of the fresh mass) rich in medium-chain fatty acids like 12:0 (50 Mol%), 14:0, and 10:0.

The Brassicaceae family provides additional examples of seeds producing endosperm oils enriched in unusual fatty acids. Low levels of omega-7 monounsaturated fatty acids like palmitoleic acid (16:1^Δ9^) and its elongated derivatives vaccenic acid (18:1^Δ11^) and paullinic acid (20:1^Δ13^) are naturally found in many plant species, but natural plant oils containing high levels of these monoenes are infrequent [63,64]. In seeds of three species in the family Brassicaceae (*B. napus*, *Camelina sativa*, and *Arabidopsis thaliana*), the proportion of omega-7 monounsaturated fatty acids accounts for a small percentage of total seed fatty acids. However, these monoenes are concentrated in the endosperm, a seed compartment of reduced size that comprises a single cell layer surrounding a large embryo structure in mature seeds [37,64,65,66,67]. In *A. thaliana* for example, monoenes of the omega-7 fatty acid series represent more than 20 Mol% of total fatty acids in the endosperm, whereas the relative proportion of these molecular species is tenfold lower in embryos (Figure 3D) [38].

Interestingly, recent development of mass spectrometry imaging instruments, including secondary-ion mass spectrometry (SIMS), desorption electrospray ionization-mass spectrometry (DESI-MS), and matrix-assisted laser desorption/ionization-mass spectrometry (MALDI-MS) have bridged the limitations to lipid-extract analysis and conventional microscopy, allowing for comprehensive metabolite detection in situ [68]. These approaches have revealed a heterogeneous distribution of triacyglycerol species in different seed tissues. For example, seeds of *R. communis* are valued for their production of oils containing up to 90% ricinoleic acid (Δ^12^-OH-18:1^Δ9^) [69,70]. In contrast with most dicot oilseeds, castor seeds are comprised of a large persistent endosperm, which accumulates most of the oil. A thin embryo consisting of an embryonic axis and two cotyledons sandwiched between the endosperm tissues also accumulates storage lipids. Fatty-acid analysis of dissected seed tissues pointed out slightly higher proportions of Δ^12^-OH-18:1^Δ9^ in the endosperm oil than in the embryo oil, even though ricinoleic acid constitutes the predominant form of fatty acids stored in the two fractions [71]. MALDI-MS imaging of *R. communis* seed sections then revealed striking differences in the distribution patterns of hydroxylated fatty-acid-containing triacylglycerols [70]. Tri-ricinolein, triacylglycerols containing a ricinoleic acid moiety at each of the three *sn*-position of the glycerol backbone [72], are enriched in the endosperm, whereas triacylglycerol molecular species containing two ricinoleic acid moieties are enriched in embryo tissues. Electrospray ionization tandem mass spectrometry (ESI-MS/MS) analysis of lipid extracts of separated endosperm and embryo tissues confirmed these differences in the distribution patterns of hydroxylated fatty-acid-containing triacylglycerols.

The comparison of oil accumulation in the embryo and in the endosperm of *Avena sativa* (oat), an unusual cereal accumulating up to 18% oil, revealed another interesting characteristic differentiating oil storage between the two zygotic tissues of the seed in this species [73,74]. Transmission electron microscopy revealed that in early maturing grains, oil bodies occur as individual uniform entities both in the embryo and in the endosperm. Whereas these oil bodies remain individualized in the late maturing embryo and in the aleurone, the oil bodies of the starchy endosperm tend to coalesce and fuse, yielding a continuous smear of oil in between the starch and storage protein components of the cell. Considering the importance of oleosins to stabilize oil bodies and impede their coalescence in seeds that undergo dehydration and rehydration [55,75,76], it is tempting to speculate that a reduced content of oil-body-associated proteins in the endosperm is responsible for this phenotype [77].

## 3. Regulation of Oil Metabolism in the Endosperm of Maturing Seeds

### 3.1. Importance of Developmental Regulations

Just as reports describing the separate analysis of oils stored in the embryo and endosperm, knowledge about the mechanisms underlying the differential regulation of oil metabolism in these two seed compartments is just emerging. From the relative positions of the two zygotic tissues within seeds, with the endosperm surrounding the embryo to varying extents, the question of a putative influence of contrasted environmental and physiological parameters on oil metabolism in these two compartments logically arises. The low permeability of maternal tissues (pod or silique walls and seed or grain integuments) for gases often leads to oxygen depletion in seeds, with oxygen concentrations steadily decreasing from the integuments to the inner embryonic tissues [25,78,79,80]. Likewise, the transmittance of light through maternal tissues is fairly low, and declines further as the depth of the tissue increases. This can potentially impact ATP and reductant production, as well as photosynthetic oxygen release in zygotic tissues of photoheterotrophic seeds relying on photosynthesis to drive oil biosynthesis [81,82,83]. A contrasted impact of such parameters on oil metabolism in the two zygotic tissues cannot be neglected, especially in large seeds. However, three-dimensional visualization of membrane phospholipids and triacylglycerols in oil-accumulating seeds has revealed very different lipid compositions in adjacent endospermic and embryonic tissues. This suggests a prevalence of tissue-specific developmental regulations of lipid metabolism [68]. In Brassicaceae, for example, the use of mass spectrometry imaging instruments has suggested a previously unappreciated role for the prokaryotic pathway in assembly of membrane lipids in the endosperm that was not observed in the embryo [84,85].

Expression studies and transcriptomic analyses performed on whole endospermic seeds and on dissected endosperm fractions [26,27,70,71,86,87,88,89] have highlighted the key role played by transcriptional regulations in the regulation of oil metabolism in the secondary zygote. All these studies have depicted a transcriptional activation of lipid biosynthesis genes concomitant with the accumulation of storage lipids in the endosperm. Genes encoding glycolytic and fatty-acid biosynthetic enzymes are usually upregulated at the onset of the maturation phase, whereas induction of genes encoding ER-associated fatty-acid-modifying and triacylglycerol-assembly enzymes is slightly delayed, just as that of oleosin-coding genes [88]. Interestingly, when Troncoso-Ponce and colleagues [86] performed a comparative transcriptional profiling of dissected seed tissues (embryos of *B. napus* and *Tropaeolum majus*, and endosperms of *R. communis* and *Euonymus alatus*), they observed that ESTs representing almost all reactions of fatty-acid synthesis were expressed with comparable stoichiometry and with consistent temporal profiles, regardless of the site of oil storage. They hypothesized that these transcriptional patterns might represent universal aspects of oilseed development within the plant kingdom, and that commons sets of core isoforms involved in storage-lipid synthesis might have been conserved throughout plant evolution.

Examination of expression profiles and transcriptomic datasets generated in different oilseed species suggests that transcriptional activation of core fatty-acid biosynthesis genes might be controlled by WRINKLED1 (WRI1) transcription factors in oil-accumulating endosperms, just as in embryonic tissues that store oil. In *A. thaliana*, *AtWRI1* is expressed in the two zygotic tissues during seed maturation [88,90]. In *E. guineensis*, three paralogous copies of *WRI1* are transcribed in the endosperm, although with different intensities [26]. However, functional evidence for the involvement of the WRIs in the activation of the core fatty acid biosynthesis pathway in endosperm tissues is still missing. Likewise, expression profiles of master regulators of seed maturation (*LEAFY COTYLEDON1* and *2*, *FUSCA3*, and *ABSCISIC ACID INSENSITIVE3*) in oil-storing endosperms suggest that they might govern oil accumulation in this tissue in a way similar to that described in embryos synthesizing reserve lipids [25,91,92]. However, these regulations remain to be confirmed and thoroughly characterized in the endosperm. To date, most of the knowledge gained in the field arose from the characterization of whole seeds or embryos of *A. thaliana* mutants, with hardly any attention given to the secondary zygote [93,94,95].

To understand how metabolic pathways and transcriptional regulatory mechanisms sharing so many characteristics can yield contrasting oil contents and compositions in adjacent endosperm and embryo tissues, a closer examination of these pathways and their transcriptional controls is necessary. In seeds of *A. thaliana*, for example, a detailed time-course analysis of oil deposition has revealed a window for oil deposition that is narrower in the endosperm than in the embryo. This biochemical pattern is correlated with an earlier decrease in the transcript abundance of fatty-acid biosynthetic genes and that of *AtWRI1* in the endosperm [88]. These observations suggested the existence of tissue-specific regulatory processes fine-tuning oil biosynthesis in the endosperm. The AtMYB118 transcription factor was thus shown to repress oil accumulation in the endosperm, contributing to a differential partitioning of reserves between the two zygotic tissues [88]. The molecular mechanism underpinning this regulatory process involves a crosstalk with master regulators of seed maturation and remains to be fully elucidated [96].

### 3.2. Transcriptional Control of Fatty-Acid Composition

The compared analysis of transcriptomic or RNAseq data obtained from dissected seed fractions has paved the way for the elucidation of the transcriptional regulations responsible for the divergent fatty compositions of endosperm oils in seeds of certain species. A differential activation of a limited subset of enzymes participating in oil metabolism usually explains these compositional changes. In *A. thaliana*, for example, recent studies have described how the specialization of endosperm tissues in omega-7 metabolism relied on the setup of a transcriptional machinery able to precisely control the spatiotemporal expression of two acyl-ACP desaturase (AAD)-coding genes [38]. *MYB115* and *MYB118*, which encode two closely related members of the MYB family of transcription factors, are transcriptionally induced at the onset of the maturation phase in the endosperm. These two MYBs were both shown to be necessary for endosperm-specific activation of *AAD2* and *AAD3* and the subsequent accumulation of omega-7 monounsaturated fatty acids in this tissue. Accordingly, the relative proportion of omega-7 is drastically reduced in the endosperm of *myb115 myb118* double mutants, as in the endosperm of *aad2 aad3* double mutants. AAD2 and AAD3 are divergent AAD isoforms that preferentially use 16:0 instead of 18:0 as substrate (Figure 1). These two Δ^9^ palmitoyl-ACP desaturases catalyze the synthesis of palmitoleic acid (16:1^Δ9^), which can be further elongated to vaccenic acid (18:1^Δ11^) in the plastid, then to paullinic acid (20:1^Δ13^) in the ER. Once synthesized, these omega-7 monoenes seem to be efficiently channeled into triacylglycerols by ‘unspecialized’ lipid biosynthetic enzymes. In this context, overexpression of *AAD2* or *AAD3* is sufficient to yield significant increase in omega-7 monoenes in both endosperm and embryo oils [64]. Interestingly, MYB115 and MYB118 thus appear to antagonistically regulate two distinct gene subcircuits that both impact storage-lipid metabolism in the endosperm. They repress the overall process of triacylglycerol accumulation (see above), while concomitantly triggering the expression of two desaturase genes responsible for increasing the proportion of unusual omega-7 monounsaturated fatty acids in this oil.

Production of medium-chain fatty acids in the seed endosperm of several species in the tribe Cocoseae also relies on tissue-specific upregulation of neofunctionalized paralogs coding for specialized biosynthetic enzymes [26]. It is generally accepted that the substrate specificity of FatA and FatB acyl-ACP thioesterases can determine to a large extent the fatty-acid composition of storage lipids [39]. All the plant species accumulating medium-chain fatty acids possess specialized acyl-ACP thioesterases of the FatB class that preferentially cleave medium-chain acyl-ACP [41]. Among the three *FatB* paralogs identified in *E. guineensis*, the level of transcription of *EgFatB3* is the most proportionally related to medium-chain fatty-acid accumulation. Massively transcribed in the endosperm, which contains 73% medium-chain fatty acids at maturity, *EgFatB3* is only moderately expressed in the embryo whose medium-chain fatty acid content is 7%, and not expressed at all in the mesocarp of the fruit, which produces oil deprived of medium-chain fatty acids [26] (Figure 1). Heterologous expression of *EgFatB3* in leaves of *Nicotiana benthamiana* confirmed that the thioesterase is able to prematurely cleave the elongating acyl-ACP in plastids. Three *FatB* genes were also identified in *C. nucifera*, a close relative of oil palm [97]. Among the three coconut FatB isoforms, CnFatB3, which shows the highest similarity with EgFatB3, is the only isoform leading to medium-chain fatty-acid accumulation in *Escherichia coli* [97] and in *A. thaliana* [98]. *CnFatB3* is highly transcribed in the endosperm, where accumulation of *CnFatB3* transcripts closely parallels that of medium-chain fatty acids [99]. Phylogenetic analyses combined with fine biochemical characterization of seed-oil composition in the family Arecaceae suggest that duplication of *FatB* genes and neofunctionalization of the *FatB3* paralog occurred in the common palm ancestor [100]. Whether the setup of transcriptional mechanisms dedicated to the endosperm-specific expression of this paralog preceded the separation into subfamilies too, or conversely, occurred independently in different palm lineages, remains unsolved.

Differential expression of genes encoding enzymes of triacylglycerol metabolism also plays an important role in medium-chain fatty-acid accumulation in endosperm oil of different species in the tribe Cocoseae. DGATs, which catalyze the final step of triacylglycerol assembly through the Kennedy pathway, exhibit variations in substrate specificity, and hence can significantly contribute to the final fatty-acid composition of plant oils [27]. *EgDGAT1-1* is induced in the endosperm of *E. guineensis* seeds and encodes a DGAT that preferentially uses medium-chain fatty acids for triacylglycerol assembly when expressed in heterologous systems such as *Yarrowia lipolytica* [101] or leaves of *N. benthamiana* [102]. These observations led to hypothesize that *EgDGAT1-1* induction may constitute a determinant of medium-chain triacylglycerol storage in the endosperm of *E. guineensis* seeds. Similarly, the *CnDGAT1* gene identified through de novo transcriptome assembly from *C. nucifera* developing endosperm was proposed to encode a DGAT with substrate preference for medium chains that may be a determinant for medium-chain triacylglycerol accumulation in the endosperm of coconut [27]. If the importance of these DGAT isoforms for medium-chain fatty-acid channeling into triacylglycerol is not questioned, it should be noted, however, that no strict correlation could be observed so far between the levels of corresponding *DGAT* transcripts in seed tissues and that of medium-chain fatty acids, in contrast to what was described for *FatB3* transcripts, for example [26]. This suggests that transcriptional activation of this enzymatic step does not control medium-chain fatty-acid accumulation in a quantitative manner. Finally, the lack of *CPT* and *PDCT* transcripts and the very low abundance of *FAD2* transcripts specifically observed in the endosperm of palm oil, together with the high transcriptional activation of *LPCAT-2* in this tissue, could explain the very low abundance of polyunsaturated fatty acids in endosperm oil, while offering mechanisms preventing incorporation of medium-chain fatty acids in membrane lipids (Figure 2) [26]. Medium-chain fatty acids are indeed known to perturb the structural integrity of membrane bilayers [103].

Whole transcriptome profiling of dissected endosperm and embryo tissues from *R. communis* seeds has provided another interesting example of contrasted transcriptional regulations targeting the pathways leading to triacylglycerol production and accounting for the heterogeneity of hydroxylated fatty acid containing triacylglycerols observed in these tissues (Figure 2). Biosynthesis of ricinoleic acid is catalyzed by ER-localized fatty-acid hydroxylases that hydroxylase 18:1^Δ9^ at the *sn*-2 position of PC [104]. Based on transcript abundance, the capacity for hydroxylation seems unlikely to account for the differences observed in relative amounts of ricinoleic acid incorporated into triacylglycerols [70]. Whereas the embryo appears to favor removal of ricinoleic acid from PC through PDAT, alternative pathways seem to be induced in the endosperm. Transcripts of *RcPLA2α* are significantly more abundant in the endosperm [70]. The phospholipase A RcPLA2α was shown to have high specificity for ricinoleic acid in yeast microsome activity assays [105], supporting the idea that ricinoleic acid is edited out from PC by this enzyme, and thus made available to enter the acyl-CoA pool for incorporation into any acyl position of triacylglycerol by acyl-CoA-dependent acyltransferases. Accordingly, several transcripts encoding LPAAT isoforms (RcLPAT2, 4, 5) are enriched in the endosperm, with RcLPAT2 exhibiting specificity toward ricinoleoyl-CoA in vitro [106]. This offers a mechanism for enriching ricinoleic acid at the *sn*-2 position of triacylglycerol. An alternative mechanism for enriching ricinoleic acid in triacyglycerols is through PDCT, which catalyzes the transfer of the phosphocholine headgroup from PC to DAG [107]. Transcripts for *RcPDCT* are elevated at significantly higher levels in endosperm tissues compared with the embryo [70]. Altogether, these observations support several complementary mechanisms that are likely to be responsible for the differential distribution of mono-, di-, and tri-hydroxylated fatty-acid-containing triacylglycerol molecular species observed in the endosperm of *R. communis*.

## 4. Biological Functions of Oils Stored in the Endosperm

### 4.1. Promoting Seed Dispersion?

Lipids stored in the oily mesocarp of fruits typically serve in the attraction of animals that consume these nutritious fruits, thus favoring animal-mediated seed dispersal, or zoochory [100,108]. For example, oily fruits of *Euterpe precatoria*, *Geonoma undata*, and *Prestoea acuminate* represent an important part of the diet of *Steatornis caripensis* (oilbird) [109], while the black agouti (*Dasyprocta fuliginosa*) is the main disperser of the very large oily fruits produced by *Mauritia flexuosa* [110]. In the fleshy mesocarp of these fruits, triacylglycerol molecules are stored in lipid droplets mostly deprived of oleosins, so that these droplets tend to fuse with one another [111]. As the fruit ripens, cell walls break down, triacylglycerols are released, and fatty acids become oxidized. The resulting soft oily mush is highly attractive to animals [112]. The observation of smears of oil in the dead endosperm cells of *A. sativa* grains raises questions about the fate and biological function of these lipids [74,113]. Most cereals, including *A. sativa*, are described as epizoochoric or anemochoric. However, granivorous birds can also eat grains and accomplish dispersal, albeit at the cost of the significant mortality of the seeds, as the muscular gizzards of the birds damage the seeds [114]. Efficient secondary dispersal of *A. sativa* grains via carnivores’ guts was also suggested, as seeds retaining the capacity to germinate were found along with feathers of birds in feces of medium-sized carnivores [115]. Whether fused oil bodies found in the endosperm of oat grains can contribute to attract granivorous birds remains unknown, however.

### 4.2. Driving Water Uptake within the Germinating Seed

Water uptake is considered as a fundamental requirement for the initiation and completion of seed germination [116]. Uptake of water by dry seeds is classically described as triphasic, with a rapid initial uptake followed by a plateau phase, then by a further increase in water uptake once germination is completed. Three-dimensional imaging of water uptake relying on noninvasive nuclear magnetic resonance (NMR) technology has revealed the inhomogeneous distribution of water in seed tissues during the imbibition process [117]. In seeds of *B. napus*, for example, water uptake appears as a highly orchestrated process that plays a key role in the transition from a dry state to active metabolism [118]. Munz and colleagues describe the germinating seed as a ‘water clock,’ the functional architecture of which predetermines the spatial and temporal setup of germination events. Seeds of *B. napus* are just a few millimeters in diameter. The entry point of water is restricted to a very small section of the integuments abutting the hilum. As imbibition progresses, water reaches neighboring regions of the integuments, hydrating them from the inside while the embryo, protected by the lipid-rich endosperm layer, stays dry. At that stage, storage lipids accumulated in the endosperm play the role of an efficient hydrophobic barrier between the embryo and the integuments. In the region close to the radicle apex, lipid deposits are partially lacking though, and this lipid gap allows the water to be further channeled directly toward the radicle tip, from whence it is distributed toward embryo cotyledons via embryonic vasculature [118]. Importantly, resumption of respiration and metabolic reactivation follows the spatiotemporal sequence of tissue rehydration [119]. In a similar manner, the uneven distribution of lipids observed in lipid-rich tissues of the poikilohydric resurrection plant *Myrothamnus flabellifolia* was presented as part of a strategy used by the plant to influence water movement, minimize transpirational water loss upon hydration, and thus cope with extreme drought [120].

### 4.3. A Source of Carbon for the Seedling

Germination is a critical period of the plant life cycle, especially in annuals, and often plays a crucial role in determining plant fitness [121]. During this process, and prior to initiation of photosynthesis in the seedling, nearly all nonparasitic angiosperms rely exclusively on reserves stored in seeds to meet two critical requirements: a source of carbon skeleton precursors and an energy source to assemble these precursors. Lipids, the principal reserve material in most seeds, fulfill these two requirements most effectively [122]. In *A. thaliana*, carbohydrates derived from endosperm lipids were unambiguously shown to be required for postgerminative seedling growth, at least in the dark [65]. Contrarily to carbon derived from embryonic reserves, carbon from endospermic reserves has to be transported to the embryo to fuel postgerminative growth [123]. Depending on the species considered, the rate of endosperm lipid mobilization can vary a lot. Lipids stored in the thin endosperm layer of *A. thaliana* seeds are mobilized within a few days [124]. The more rapid mobilization of endosperm oil compared with embryo oil in this species is consistent with both the contrasted sizes of the lipid pools stored in the two seed fractions [88] and the sequence of tissue rehydration described in Brassicaceae seeds (see above). In contrast, lipids stored in the large endosperm of coconut seeds are remobilized more tardily. Remobilization is spread over several months, long after the plantlet has become photosynthetic [61].

**Figure 4:**
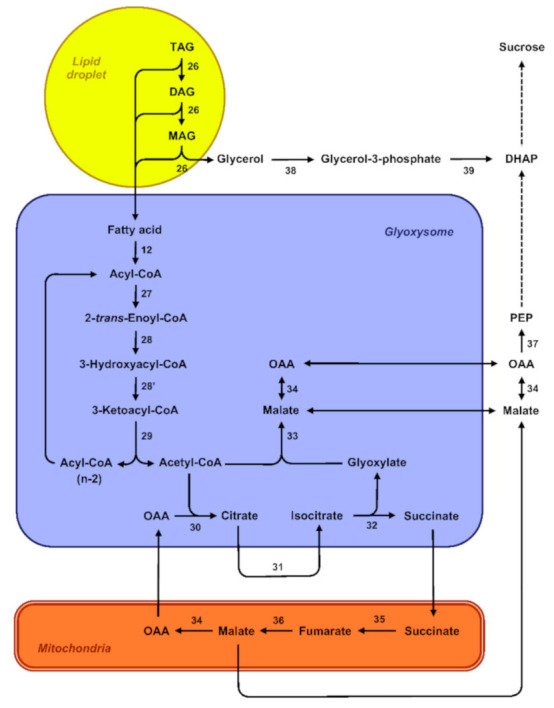
Overview of oil metabolism in germinating seeds. The scheme depicts the different pathways involved in the mobilization of storage lipids in germinating seeds. 12, long-chain acyl-CoA synthetase; 26, triacylglycerol lipase; 27, acyl-CoA oxidase; 28, multifunctional protein 2-*trans*-enoyl-CoA hydratase; 28′, multifunctional protein 1,3-hydroxyacyl-CoA dehydrogenase; 29, 3-ketoacyl-CoA thiolase; 30, citrate synthase; 31, aconitase; 32, isocitrate lyase; 33, malate synthase; 34, malate dehydrogenase; 35, succinate dehydrogenase; 36, fumarase; 37, phosphoenolpyruvate carboxykinase; 38, glycerol kinase; 39, glycerol-3-phosphate dehydrogenase. Abbreviations: CoA, coenzyme A; DAG, diacylglycerol; FA, fatty acid; MAG, monoacylglycerol; TAG, triacylglycerol.

From a theoretical perspective, degradation of reserve lipids can either involve autophagy-related pathways, referred to as lipophagy, or lipases in the case of cytosolic lipolysis [125]. During the germination of *R. communis* seeds, expression patterns of autophagy-related *RcATG* genes were associated with the degradation of storage lipids. What is more, observations of endosperm cells by electron microscopy revealed lipid droplets swallowed by vacuoles. This suggests that autophagy directly participates in mediating the decomposition of lipid reserves via the microlipophagy pathway, a nonselective degradation process in which degradative materials are directly engulfed by vacuoles [126]. Except in this recent report, the role of autophagy in the mobilization of storage lipids during the post-germinative phase has hardly been documented, and the lipolysis pathway is thought to prevail in most germinating oilseeds. In this pathway, degradation of the oleosins coating the lipid droplets is usually considered as a prerequisite for the mobilization of triacylglycerols [127]. Then, lipases, which are interfacial enzymes, successively cleave tri-, di-, and monoacylglycerol molecules into free fatty acids and glycerol (Figure 4) [128]. Free fatty acids are then degraded through the ß-oxidation and glyoxylate cycles and subsequently converted into sugars [129]. ß-oxidation converts fatty acids to acetyl-CoA in specialized peroxisomes called glyoxysomes [130]. Fatty-acid substrates have to be activated by esterification with coenzyme A before entering the ß-oxidation spiral. The repeated cleavage of acetate units from the thiol end of the fatty acyl-CoA after oxidative attack at the C-3 or ß-carbon position involves three enzymes: acyl-CoA oxidase (ACX) oxidizes acyl-CoA to 2-*trans*-enoyl-CoA, multifunctional protein (MPF) exhibits both 2-*trans*-enoyl-CoA hydratase and 3-hydroxyacyl-CoA dehydrogenase activities, and 3-ketoacyl-CoA thiolase (KAT) catalyzes the thiolytic cleavage of 3-ketoacyl-CoA to acyl-CoA (Cn-2) and acetyl-CoA [131]. Fatty acids found in storage oils often have unsaturated bonds in the *cis*-configuration at even-numbered carbons or unsaturated bonds at odd-numbered carbons that cause a metabolic block of the ß-oxidation pathway if only the core set of enzymes is considered. Their degradation consequently requires auxiliary enzymes to convert these double bonds and generate the 2-*trans*-enoyl-CoA intermediate substrate of MPF activity [129]. Acetyl-CoA derived from fatty-acid ß-oxidation is further metabolized via the glyoxylate cycle that involves two enzymes unique to this cycle, namely isocitrate lyase and malate synthase. Three additional enzymes also participate in the TCA cycle: citrate synthase, aconitase, and malate dehydrogenase. This cycle is distributed between different cell compartments and likely involves a very close coordination between glyoxysomes and mitochondria [132]. Four-carbon compounds from the glyoxylate cycle can be converted into hexoses by gluconeogenesis and subsequently converted into sucrose. In *A. thaliana* endosperm, the cytoplasmic phosphoenolpyruvate carboxykinase PCK1 catalyzes the critical first step in gluconeogenesis [65].

**Figure 5:**
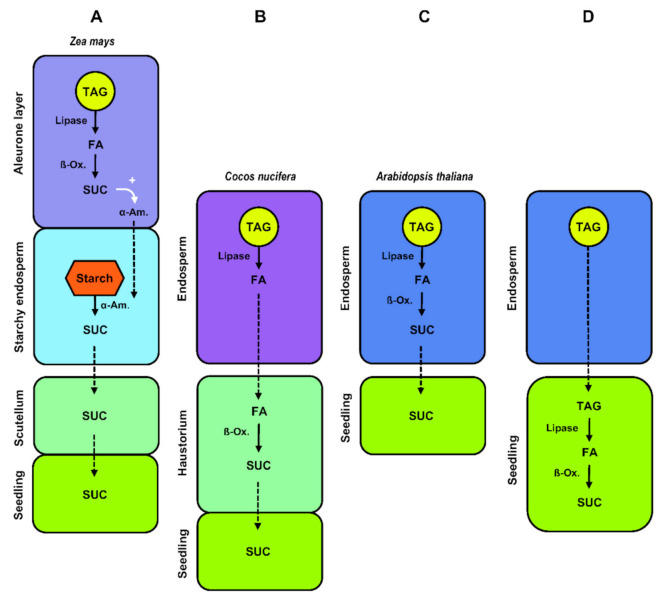
Oil mobilization in germinating seeds. The schemes illustrate different strategies for the mobilization of storage lipids and the allocation of carbon to the seedling. (**A**). In the aleurone layer of cereal grains, lipid mobilization supports the synthesis of α-amylases that are secreted into the dead starchy endosperm to mobilize starch, releasing nutrients that are absorbed by the scutellum. (**B**). In non-cereal monocots in the family Aracaceae, fatty acids released from triacylglycerol by lipases in the endosperm are thought to be transported to the haustorium, where enzymes of ß-oxidation are located. (**C**). In seeds of oleaginous dicots, triacylglycerol mobilization and gluconeogenesis take place in the endosperm and sugars are transported to the seedling. (**D**). Although this hypothesis remains to be validated, direct supply of triacylglycerol from the endosperm to the seedling was also proposed. Abbreviations: α-Am, α-amylase; ß-ox., ß-oxidation; FA, fatty acid; SUC, sucrose; TAG, triacylglycerol.

From the biochemical studies of lipid remobilization carried out in the endosperm of germinating oilseeds in dicots, it was assumed that complete conversion of triacylglycerols to sugars took place in the endosperm [133,134,135], implying the existence of active transport systems translocating sugars from the endosperm to the seedling (Figure 5C). In agreement with this model, a transcriptomic approach aimed at studying the expression of genes in the endosperm of germinating *A. thaliana* seeds revealed that all the major characterized fatty-acid ß-oxidation transcripts were highly expressed in this tissue shortly after radicle emergence, just as transcripts encoding enzymes involved in gluconeogenesis and sugar transporters [136]. What is more, the characterization of *A. thaliana pck1* mutants firmly established that the export of carbon from the endosperm to the embryo during skotomorphogenesis was dependent on endosperm gluconeogenesis [65]. Based on current knowledge of lipid remobilization in seeds of dicots, it is not clear whether alternative pathways involving the direct export of lipids from the senescing endosperm to the embryo also exist. In *Euphorbia lagascae*, different transcripts encoding lipid transfer proteins (LTP) were detected in endosperm and embryo cotyledons of germinating seeds, with those encoding the ElLTP2 isoform forming a concentration gradient in the endosperm, with higher amounts in the inner regions close to the cotyledons, and lesser amounts in the outer regions of the endosperm [137]. Lipid-transfer proteins are characterized by their structure and ability to enhance the in vitro exchanges of various polar lipids between different membranes [138], but the in vivo functions of these proteins remain disputed [139]. From the observations made in *E. lagascae* seeds*,* it was postulated that lipid-transfer proteins might be involved in the recycling of endosperm lipids and their transfer to the adjacent embryo cotyledons (Figure 5D) [140]. However, functional evidence is still lacking to unambiguously support this theory.

In non-cereal monocots in the family Arecaceae like *Phoenix dactylifera* (date palm), *E. guineensis*, and *C. nucifera*, the mobilization of reserve compounds stored in the endosperm is a complex process involving the development of a highly specialized absorptive and storage organ called the haustorium, which develops from the cotyledonary blade of the embryo [61]. The connection of the haustorium with the vegetative axis of the seedling by vascular bundles observed in *Acrocomia aculeata* (macaw palm) [141] or *P. dactylifera* [142] denotes the functions of this organ in absorption and transport. The growing haustorium expands extensively as the endosperm disappears until it completely fills the seed. The process of endosperm breakdown is confined to a narrow digestion zone directly adjacent to the invaginated surface of the haustorium, and continues at a rate commensurate with haustorium development [143,144]. The position of the endosperm digestion zone provides structural evidence that the haustorium acts as a regulator of reserve mobilization in this tissue (Figure 5B) [145]. In germinating *E. guineensis* seeds, the release of free fatty acids from triacylglycerol catalyzed by lipases is thought to occur in the endosperm. Free fatty acids might then be absorbed by the haustorium, where the ß-oxidation enzymes are located [146,147]. This transport remains uncharacterized though. Fatty acyl-CoA esters produced by fatty acyl-CoA synthetase have been proposed as a form of transport [148]. Even though evidence is lacking, the hypothesis of a direct import of triacylglycerol possibly released in the extracellular medium surrounding the haustorium after collapse and breakdown of endosperm cells, followed by haustorium-located lipolysis, cannot be completely ruled out [141,149]. Sugars generated in the haustorium after peroxisomal degradation of fatty acids via ß-oxidation and gluconeogenesis can be directly transported to the growing axis of the seedling or converted to starch and temporarily stored in the haustorium as described in *Butia capitata* [144,150], *A. aculeata* [141], *C. nucifera* [143], and *E. guineensis* [151].

Lipid mobilization in the endosperm of germinating cereal grains follows different pathways and fulfills different functions, depending on the site of oil storage. It is generally believed that the oil stored in the aleurone layer of cereals is mobilized early during germination, before the arrival of sugars derived from starch degradation in the starchy endosperm (Figure 5A) [113,152]. Aleurone cells metabolize free fatty acids released from neutral lipids via ß-oxidation and the glyoxylate cycle [153,154]. These cells then synthesize and secrete a battery of hydrolytic enzymes (principally α-amylases and proteinases) that are secreted into the dead starchy endosperm to mobilize starch and storage protein reserves, releasing nutrients that are absorbed by the scutellum and transported to the growing embryo. Shortly thereafter, aleurone cells undergo programmed cell death. Gluconeogenesis of lipid reserves within aleurone cells is thought to support the synthesis of these hydrolytic enzymes, thus contributing indirectly to the supply of sugars and amino acids for the seedling.

Since the starchy endosperm of cereal grains goes through programmed cell death upon maturation, this tissue lacks glyoxysomes and therefore cannot serve as a site for free-fatty-acid degradation in germinating grains, even when large amounts of neutral lipids are stored in this endosperm tissue as in *A. sativa* [15,113]. Lipases secreted from the scutellum and possibly the aleurone layer into the endosperm might release free fatty acids from triacylglycerol molecules [155,156] before the scutellum absorbs these fatty acids. In the scutellum, fatty acids are most probably degraded through ß-oxidation and subsequently converted into sugars that can be further transported to the embryo through the scutellar vasculature [157]. Since oil in the endosperm of *A. sativa* can come in close contact with epithelium cells of the scutellum during germination, the possibility that endosperm triacylglycerol is taken up directly by the scutellum and immediately degraded into free fatty acids by lipases in this tissue cannot be completely excluded, even though evidence for such triacylglycerol transport is lacking [113].

While an important research effort has been directed toward the elucidation of the regulatory mechanisms controlling seed dormancy and germination in dicots and monocots [158,159,160], the regulation of post-germinative events such as oil mobilization has been less well characterized. In the light of current knowledge, gibberellins (GAs) act as a diffusible signal from the embryo that induces triacylglycerol mobilization in the endosperm of dicots [65,161] and in aleurone cells of cereals [153,162,163,164]. In *H. vulgare* aleurone, however, a survey for the activity of the glyoxylate enzyme isocitrate lyase in different cultivars revealed contrasted requirements for GAs for the induction of the enzyme, possibly as a result of selective breeding to alter seed dormancy [28]. Abscisic acid (ABA) efficiently antagonizes the effect of GAs and represses the flux of carbon from oil to sugars in *H. vulgare* aleurone [28]. In contrast, induction of lipid-reserve-mobilization genes in the endosperm of *A. thaliana* cannot be blocked by ABA [65]. The ABA insensitivity of lipid breakdown in *A. thaliana* endosperm can be attributed specifically to the lack of expression of the *ABSCISIC ACID INSENSITIVE4* (*ABI4*) gene, whose expression is confined to the embryo [136]. *ABI4* encodes an AP2-ERF transcription factor that behaves as an enhancer in the ABA signal transduction pathway [165].

### 4.4. Mobilization of Storage Lipids and ROS Signaling

Mobilization of storage lipids is associated with an intense production of reactive oxygen species (ROS) [166]. For example, lipoxygenase-catalyzed oxygenation of storage lipids was shown to participate in lipid mobilization during germination in various species [167]. The products of this oxygenation reaction are highly reactive hydroperoxides that are normally reduced to the corresponding hydroxyl fatty acyl chains by a reductase, before being hydrolyzed by lipases and further catabolized by ß-oxidation. Then, breakdown of fatty acyl chains to acetyl-CoA in the glyoxysome by ß-oxidation also generates ROS, regardless of the type of fatty acyl chain processed. The first enzyme in this pathway, acyl-CoA oxidase, is a flavin-containing enzyme that reduces O_2_ directly to H_2_O_2_. The H_2_O_2_ produced during ß-oxidation is efficiently converted to O_2_ and H_2_O by highly active catalases located in the glyoxysome [130]. When the amounts of catalases and other ROS-metabolizing enzymes decrease, however, concentrations of ROS can increase. H_2_O_2_ is highly mobile within the cell and is a versatile compound that can have toxic or signaling effects in seeds [168]. If several studies have shown that ROS can interplay with the hormonal signaling pathways involving ABA and GAs in the control of the transition from a dormant to a non-dormant state [169,170], it seems unlikely that ROS production associated with reserve lipid mobilization participates in this regulation, given that this metabolic process is regarded as a post-germinative event. By contrast, ROS produced during lipid mobilization have been implicated in the programmed cell death of aleurone cells in cereal grains [164]. Once aleurone cells have completed their role as a secretory tissue, synthesizing and secreting hydrolytic enzymes into the starchy endosperm, a decline in glyoxysomal catalase precedes death of aleurone cells, and this compromised capacity to metabolize ROS may contribute to an increase in cellular oxidative stress [171]. Whether ROS play a role in the death program as switch for a signal transduction cascade that initiates aleurone programmed cell death or by contributing to the execution phase remains unclear. Endosperm cells are known to be able to sense environmental changes (e.g., light) and synthesize signals for the purpose of regulating different aspects of seed germination [172]. Interestingly, it was proposed that the effects of blue and UV lights on aleurone cell death could be mediated via flavin-containing enzymes such as acyl-CoA oxidase [164].

## 5. Conclusions

Oil storage in the endosperm of seeds is widespread among angiosperms and can take different forms. Reserve lipids play different pivotal roles during seed germination and contribute to the success of the establishment of the young seedling. Despite the importance of endosperm oils in seed biology, our knowledge of the specificities of oil metabolism in this tissue remains largely incomplete. The emergence and continuous improvement of analytical techniques allowing the evaluation, within a spatial context, of gene activity on one side, and lipid metabolism on the other side, provide us with a unique opportunity to separately examine different seed cell types and support studies of endosperm biology, even in small seeds. A key to understanding lipid metabolism in the endosperm is to define the regulatory circuitry that governs genes differentially expressed in this tissue. Regarding the mobilization of oil in the endosperm of germinating seeds, it appears that the form in which carbon is supplied to the seedling (either as intact triacylglycerol, as free fatty acids released from triacylglycerol by lipases, or as sugars through free-fatty-acid degradation by ß-oxidation with subsequent gluconeogenesis, or a combination of these alternatives) and the corresponding transport processes remain uncertain in many species. Stimulating discoveries in this research field are expected not only to increase our understanding of the basics of seed biology, but also to impact the improvement of seed varieties.
